# Human Umbilical Vein Endothelial Cells Form a Network on a Hyaluronic Acid/Gelatin Composite Hydrogel Moderately Crosslinked and Degraded by Hydrogen Peroxide

**DOI:** 10.3390/polym14225034

**Published:** 2022-11-20

**Authors:** Kelum Chamara Manoj Lakmal Elvitigala, Wildan Mubarok, Shinji Sakai

**Affiliations:** Department of Materials Engineering Science, Graduate School of Engineering Science, Osaka University, Toyonaka 560-8531, Japan

**Keywords:** composite hydrogel, hyaluronic acid, enzymatic crosslinking, degradation, in-vitro angiogenesis model

## Abstract

The study of the capillary-like network formation of human umbilical vein endothelial cells (HUVECs) in vitro is important for understanding the factors that promote or inhibit angiogenesis. Here, we report the behavior of HUVECs on the composite hydrogels containing hyaluronic acid (HA) and gelatin with different degrees of degradation, inducing the different physicochemical properties of the hydrogels. The hydrogels were obtained through horseradish peroxidase (HRP)-catalyzed hydrogelation consuming hydrogen peroxide (H_2_O_2_, 16 ppm) supplied from the air, and the degradation degree was tuned by altering the exposure time to the air. The HUVECs on the composite hydrogel with intermediate stiffness (1.2 kPa) obtained through 120 min of the exposure were more elongated than those on the soft (0.4 kPa) and the stiff (2.4 kPa) composite hydrogels obtained through 15 min and 60 min of the exposure, respectively. In addition, HUVECs formed a capillary-like network only on the stiff composite hydrogel although those on the hydrogels with comparable stiffness but containing gelatin alone or alginate instead of HA did not form the network. These results show that the HA/gelatin composite hydrogels obtained through the H_2_O_2_-mediated crosslinking and degradation could be a tool for studies using HUVECs to understand the promotion and inhibition of angiogenesis.

## 1. Introduction

The capillary network, composed of vascular endothelial cells, is essential for nutrient and gas exchanges between blood and tissue. In addition, capillary network formation is essential for tumor growth [1,2]. Therefore, studies dealing the capillary network formation have been intensively conducted both in vivo and in vitro [3,4,5]. Various hydrogels enabling the network formation of vascular endothelial cells have been developed for studies in vitro [6,7]. Hydrogels are water-swollen three-dimensional networks of polymers. To provide mechanical support as well as the chemical environment for cell adhesion sites, cell remodeling, nutrient support, and cell responsive remodeling, hydrogels are widely used in tissue engineering technologies as transient artificial extracellular matrix substitutes [8,9].

The physicochemical properties of the extracellular matrix (ECM) are known to modulate the cell functions such as adhesion and proliferation [10,11]. Numerous reports have been published on the effect of chemical modifications of the ECM to control endothelial cell behavior. Rumiana et al. reported the angiogenic potential of the endothelial cell on gelatin-based hydrogel by controlling the growth factor release by electrical stimulation [12]. The report by Ying et al. showed that the co-delivery of growth factor by gelatin hydrogel promotes angiogenesis compared with growth factors applied alone [13]. Derek et al. reported that hyaluronidase is stimulated in endothelial colony-forming cells through hyaluronic acid (HA)-specific receptors for cord-like structures on HA hydrogels [14].

In addition, ECM stiffness also plays a vital role in cell functions. For example, Nelson et al. demonstrated that gelatin methacryloyl hydrogel stiffness was controlled by enzymatic degradation to analyze the stiffness-dependent vascular formation [15]. However, the effect of chemical and physical properties together on the behavior of endothelial cells has been paid less attention. Thus, here, we present a novel method to control endothelial cell behavior using both physical and chemical properties of artificial ECM in gelatin and HA-based hydrogel.

Gelatin- and HA-based hydrogels have been widely utilized as artificial ECM in vitro to mimic the characteristics, properties, and good biocompatibility. Gelatin is a well-known biodegradable and low immunogenic polymer that allows Arg-Gly-Asp (RGD)-mediated cell adhesion. In addition, gelatin-based hydrogels can deliver growth factors such as basic fibroblast growth factors (bFGF), which are vital in angiogenesis [13]. HA is a widely utilized non-sulfated glycosaminoglycan found abundantly in the human body. Specific activities such as high-molecular-weight HA (HMW-HA) are crucial for wound healing [16]. In addition, HA can interact with different receptors on the cell surface and activate distinct cellular signaling. Among them, CD44 and RHAMM are identified as the most relevant for inflammation and cancer [17].

The molecular weight of HA can differently influence the activation of the cell surface receptors. Fragments of HA, called hyaluronan oligosaccharides, which promote angiogenesis can be obtained through enzymatic degradation [18]. The interaction between the HA fragments and CD44 receptors stimulates angiogenesis [19]. In contrast, gelatin and HA are important materials for endothelial cell functions, including angiogenesis. When designing an artificial ECM for tissue engineering applications, it should allow cell adhesion, proliferation, and differentiation with the same characteristic properties as the native. The scaffolds composed of HA alone are not a promising from a viewpoint of cell adhesion. The scaffolds composed of HA and integrin-binding adhesive peptides can promote cell adhesion and proliferation. Therefore, in this study, we mixed gelatin and HA to make cell adhesive composite hydrogel with modification of both polymers with phenol groups through tyramine substitution (Gelatin-Ph/HA-Ph) for the in vitro study of endothelial cell behavior.

In the present study, we used gelatin and HA derivatives both possessing phenol groups, which allow enzymatically catalyzed oxidation of phenolic hydroxyl groups, resulting in polyphenolic crosslinking at the aromatic ring through C-C and C-O coupling. To obtain polymers crosslinking for hydrogel preparation, a variety of methods have been reported, such as electron beam [20] or ultraviolet light [21] and chemical crosslinking with glutaraldehyde [22]. Due to the limitation of the above methods, such as low mechanical stability in irradiation crosslinking and cytotoxicity in chemical crosslinking, in this study, we utilized horseradish peroxidase (HRP)-mediated crosslinking to obtain Gelatin-Ph/HA-Ph composite hydrogel. HRP is a well-known biocompatible heme protein that catalyzes the conjunction of phenolic derivatives in the presence of hydrogen peroxide (H_2_O_2_).

**Figure 1:**
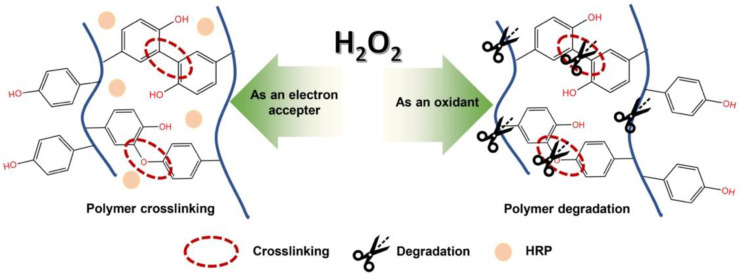
Schematic illustration of the H_2_O_2_-mediated crosslinking of phenolic groups on polymer chains catalyzed by HRP and degradation of the polymer chains.

Apart from the action of H_2_O_2_ as an electron acceptor during the crosslinking reaction, it has the potential to oxidize the polymer molecules (Figure 1). In addition, H_2_O_2_ is generally utilized for degrading various polymers, including HA and gelatin, through oxidative cleavage of the bond [23,24,25]. By considering these functions of H_2_O_2_, the physical and chemical properties of the hydrogel can be controlled by adjusting the reaction time with H_2_O_2_. Previously, we reported that the stiffness of the Gelatin-Ph- and HA-Ph-based hydrogels can be controlled by tuning air containing H_2_O_2_ exposure time [26,27,28]. In this study, we studied the behavior of human umbilical vein endothelial cells (HUVECs) on the Gelatin-Ph/HA-Ph hydrogels obtained by tuning the exposure time to H_2_O_2_.

## 2. Materials and Methods

### 2.1. Materials

Gelatin (type B from bovine skin, 226 g Bloom), basic fibroblast growth factor (bFGF), and endothelial growth factor (EGF) were purchased from Sigma-Aldrich (St. Louis, MO, USA). HA-Ph and Alginate-Ph containing 8 Ph groups per 100 repeat units were gifted from Nagase ChemteX (Osaka, Japan). Aqueous H_2_O_2_ (31% w/w), *N*-hydroxysuccinimide, dimethylformamide, 3-(4-hydroxyphenyl) propionic acid, 4% *w/v* paraformaldehyde in PBS, catalase (bovine liver), collagenase, hyaluronidase, and horseradish peroxidase (HRP) were purchased from FUJIFILM Wako Pure Chemical (Osaka, Japan). The calcein-AM and propidium iodide were obtained from Nacalai Tesque Inc. (Kyoto, Japan) and Dojindo (Kumamoto, Japan), respectively. 1-Ethyl-3-(3-dimethylaminopropyl)carbodiimide and Endothelial Basal Medium MCDB107 were obtained from Peptide Institute (Osaka, Japan). Gelatin type B possessing phenol groups was synthesized based on the previously developed protocol [29]. Briefly, dimethylformamide, 3-(4-hydroxyphenyl) propionic acid was activated using 1-ethyl-3-(3-dimethylaminopropyl)carbodiimide and *N*-hydroxysuccinimide. Then, gelatin was added and stirred for 20 h in DMF buffer solution (pH 4.2). After reacting for 20 h, unreacted 3-(4-hydroxyphenyl) propionic acid was removed from the reaction mixture by dialyzing the solution in dH_2_O. UV–vis spectrometry (UV-2600, Shimadzu, Kyoto, Japan) and NMR spectroscopy (JNM ECS400, JEOL, Tokyo, Japan) were used to confirm that the phenolic group had been introduced to the Gelatin-Ph (Figure S1 from the Supplementary Materials) [29].

### 2.2. Mechanical Property Measurement

The stiffness of the synthesized hydrogels was determined in terms of Young’s modulus using a material tester (EZ-SX, Shimadzu, Kyoto, Japan). First, composite hydrogel (HA-Ph/Gelatin-Ph) was obtained by exposing the solution of 3.0 wt% Gelatin-Ph, 0.5 wt% HA-Ph dissolved in PBS (pH 7.4), and HRP (1 U/mL) to air containing 16 ppm H_2_O_2_ for 15, 60, and 120 min. Then, each composite hydrogel was compressed at 6 mm/s using an 8 mm probe. Next, Young’s modulus was calculated to determine the stiffness of prepared hydrogel using the linear compression strain of 1–10% of the stress–strain curve. The same experimental steps were followed for the preparation and mechanical property measurements for 3.0 wt% Gelatin-Ph/0.5 wt% Alginate-Ph, 3 wt% Gelatin-Ph, and 3.25 wt% Gelatin-Ph hydrogels.

### 2.3. Enzymatic Degradation

The composite hydrogels (Gelatin-Ph/HA-Ph) were soaked in PBS for one day to reach an equilibrium state. Then, the composite hydrogels were soaked in a mixture of hyaluronidase (1 mg/mL) and collagenase (1 mg/mL) in Dulbecco’s Modified Eagle’s Medium (DMEM). Next, the time required for the total decomposition of hydrogel was measured.

### 2.4. Molecular Weight Analysis

PBS containing 3.0 wt% Gelatin-Ph and 0.5 wt% HA-Ph were separately exposed to air containing 16 ppm H_2_O_2_ for 15 min, 60 min, and 120 min, respectively. The molecular weights of resultant polymers in the solutions were analyzed using high-performance liquid chromatography (HPLC) (LC-20AD, Shimadzu, Kyoto, Japan) based on an intensity–time curve (Figure S2). The unexposed stock solutions of both polymers were used as the control, and the molecular weight of each sample was calculated from the calibration curve of polyethylene glycol standards.

### 2.5. Scanning Electron Microscope Observation of Freeze-Dried Hydrogels

Specimens of freeze-dried hydrogels were prepared based on the freeze-extraction method [30]. Briefly, 1 mL of PBS containing 3.0 wt% Gelatin-Ph/0.5 wt% HA-Ph and 1 U/mL HRP was added to a polydimethylsiloxane (PDMS) mold (diameter: 8 mm, height: 4 mm). Air containing H_2_O_2_ was then exposed for 15, 60, and 120 min. The resultant hydrogel was frozen at −80 °C. The frozen specimens were immersed in 70% and 100% ethanol in sequence at −30 °C for 10 h and subsequently vacuum dried. Then, the cross sections of the resultant specimens coated with a thin layer of gold were observed using a field emission scanning electron microscope (SEM, JCM-6000plus, JEOL, Tokyo, Japan) with an acceleration voltage of 15 kV.

### 2.6. Cell Culture

Human Umbilical Vein Endothelial Cells (HUVECs) were obtained from RIKEN Cell Bank (Ibaraki, Japan). The HUVECs were cultured in MDCB107 medium consisting of fetal bovine serum (10 v/v%) and supplemented with the 10 ng/mL of basic fibroblast growth factor (bFGF) and 10 ng/mL of endothelial growth factor (EGF) through passage 6. Cell culture was conducted in a humidified incubator supplied with 5% CO_2_ at 37 °C.

### 2.7. Cell Viability and Adhesion

The composite hydrogel (Gelatin-Ph/HA-Ph) was prepared in a 6 well-plate by exposing the air containing H_2_O_2_ for 15–120 min to 1 mL of 3.0 wt% Gelatin-Ph, 0.5 wt% HA-Ph dissolved in PBS and HRP (1 U/mL) solution. The unreacted H_2_O_2_ was removed from the hydrogel using the growth medium containing catalase (1 mg/mL). Following overnight incubation in catalase, hydrogels were washed thoroughly several times using both PBS and MDCB107 medium. HUVECs were then cultured on the polystyrene cell culture plate or hydrogel at 4.0 × 10^3^ cells/cm^2^. HUVECs viability was analyzed based on our previously reported method [28]. HUVECs adhesion to the substrates was analyzed based on the cell morphological parameters, including aspect ratio (ratio between cell length and width), and area, were calculated for the morphology analysis using ImageJ (1.53f51, NIH, Bethesda, MD, USA). Previous reports in the literature mentioned that calcein-AM, which stained the cell cytoplasm, could be used to analyze the morphological parameters. Therefore, in this study, we used the calcein-AM staining for the morphological analysis [31,32].

### 2.8. HUVECs Network Formation

HUVECs network formation analysis was conducted on the hydrogels according to the previously reported protocol [33]. First, 3 wt% Gelatin-Ph, 0.5 wt% HA-Ph, and HRP (1 U/mL) were mixed in PBS and put into a 6-well plate (1 mL/well). Then, the polymer solution was exposed to the air containing H_2_O_2_ (16 ppm) for 15–120 min. Subsequently, 1 mL of catalase dissolved in MCDB107 medium was added to fabricated hydrogel and incubated overnight at 37 °C in humidified incubator supplied with 5% CO_2_. After incubation overnight, the cells with 70% confluent were trypsinized and seeded on the Gelatin-Ph/HA-Ph hydrogel at 4.0 × 10^4^ cells/cm^2^ [33]. HUVECs were cultured in an MCDB107 medium containing 10 ng/mL bFGF, 10 ng/mL EGF, and 2 v/v% FBS. The HUVECs network formation was observed using an optical microscope (OLYMPUS IX71, Tokyo, Japan) at 20 h of post culture. Parallel experiments were conducted on the 3 wt% Gelatin-Ph/0.5 wt% Alginate-Ph, 3 wt% Gelatin-Ph, 3.25 wt% Gelatin-Ph hydrogel, and cell culture dish following the same experimental procedure.

### 2.9. Statistical Analysis

Microsoft^®^ Excel^®^ (Microsoft Corp., Redmond, WA, USA) 2019 version 1808 was used for all data analysis. Statistical analyses were performed using one-way analysis of variance (ANOVA). A post hoc *t*-test was conducted using Tukey HSD, and a *p*-value of <0.05 was considered significantly different.

## 3. Results and Discussion

### 3.1. Properties of Gelatin-Ph/HA-Ph Hydrogels

**Figure 2:**
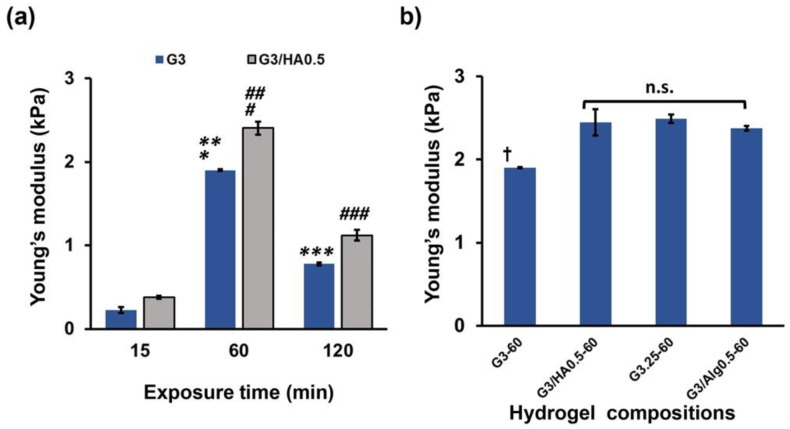
(**a**) Effect of the air containing 16 ppm H_2_O_2_ exposure time on the stiffness of G3 and G3/HA0.5 hydrogels. (**b**) Effect of the composition of polymers on the stiffness of hydrogels obtained by applying air containing H_2_O_2_ for 60 min. (G3-60, G3/HA0.5-60, G3.25-60, and G3/Alg0.5-60). Bars: S. E. (*n* = 3). * *p* < 0.01, ^#^ *p* < 0.01, 15 min compared to 60 min; ** *p* < 0.01, ^##^ *p* < 0.01, 60 min compared to 120 min; *** *p* < 0.01, ^###^ *p* < 0.01, 15 min compared to 120 min; ^†^ *p* < 0.01, G30-60 compared to G3/HA0.5-60, G3.25-60, and G3/Alg0.5-60; n.s., *p* > 0.05. Tukey HSD.

Firstly, we examined the influence of the exposure time to the air containing 16 ppm H_2_O_2_ (15, 60, and 120 min) on the stiffness of the hydrogels after confirming the hydrogelation within 1 min of the exposure for all the compositions shown in Table 1. As shown in Figure 2a, Young’s modulus increased with H_2_O_2_ exposure time from 15 to 60 min, and the values decreased with a further increase of the time to 120 min both in G3 and G3/HA0.5 hydrogels. The values detected for G3-60 and G3/HA0.5-60 after exposure to the air containing H_2_O_2_ for 60 min were 1.90 kPa and 2.40 kPa, respectively. These values were about 8- and 2-times larger than those detected for the specimens obtained through 15 and 120 min of exposure, respectively, at the same composition. The air containing H_2_O_2_ exposure time-dependent change of the hydrogel mechanical properties is consistent with the results in the literature [26,28]. The hydrogel stiffness increase with an increase of H_2_O_2_ exposure time from 15 to 60 min was explained by the increase of the crosslinking between Ph groups through the progression of HRP-catalyzed reaction [27]. On the other hand, the hydrogel stiffness decrease with an increase in the exposure time from 60 to 120 min was explained by the HRP inactivation and polymer degradation by H_2_O_2_ [26].

**Figure 3:**
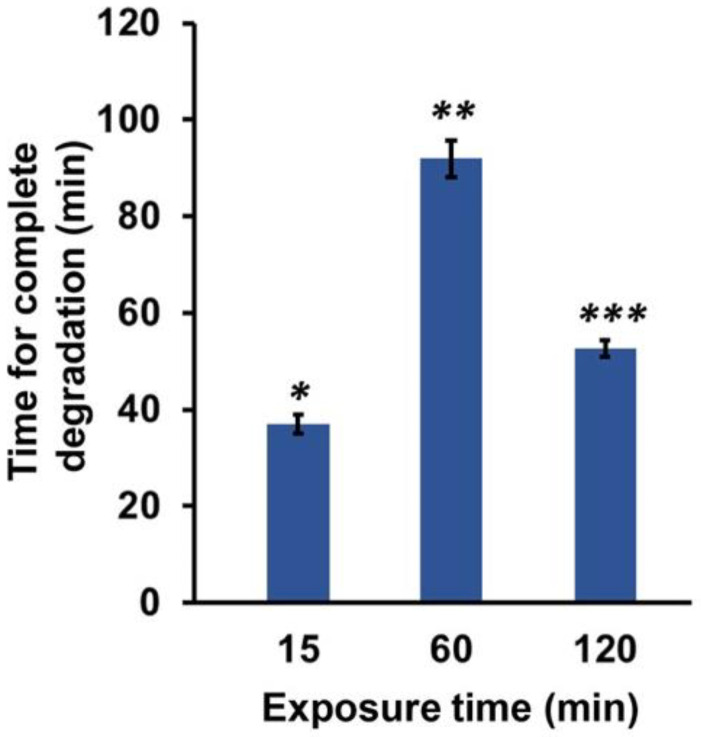
Enzymatic degradation of Gelatin-Ph/HA-Ph hydrogel by a mixture of hyaluronidase (1 mg/mL) and collagenase (1 mg/mL). The composite hydrogel was prepared by exposing air containing H_2_O_2_ for 15–120 min into PBS containing 3 wt% Gelatin-Ph/0.5 wt% HA-Ph and 1 U/mL HRP. Bar: S.E. (*n* = 3). * *p* < 0.005, 15 min compared to 60 min; ** *p* < 0.01, 60 min compared to 120 min; *** *p* < 0.01, 15 min compared to 120 min. Tukey HSD.

Furthermore, the effect of the air containing H_2_O_2_ exposure time on the degradability of the composite hydrogels were evaluated by measuring the time required for total degradation of the G3/HA0.5 hydrogel using hyaluronidase and collagenase as shown in Figure 3. The stiff hydrogel (G3/HA0.5-60) required the longest time (92 min) to be degraded, due to the high crosslinking formation compared to the soft and intermediate stiff hydrogels (G3/HA0.5-15 and G3/HA0.5-120, respectively).

**Figure 4:**
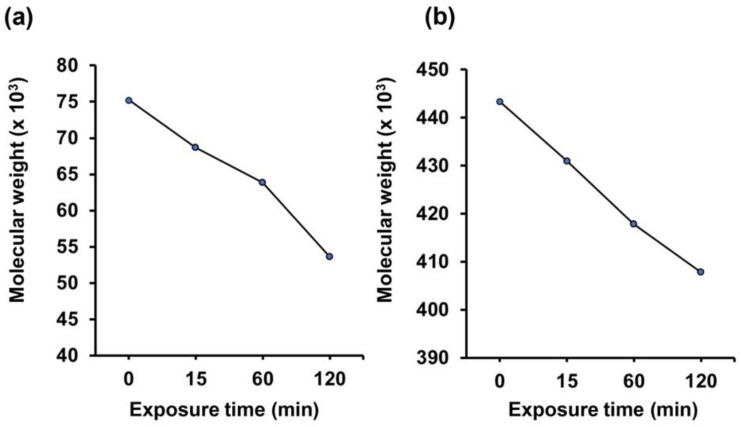
Effect of the exposure time to the air containing 16 ppm H_2_O_2_ on the molecular weights of (**a**) Gelatin-Ph and (**b**) HA-Ph.

As shown in Figure 4, the molecular weights of both Gelatin-Ph and HA-Ph decreased with extending the H_2_O_2_ exposure time. The higher stiffness of G3/HA0.5 hydrogels than G3 hydrogels at each exposure time was caused by the denser polymer network due to the higher polymer concentration. The stiffness of the hydrogels prepared from 3.25 wt% Gelatin-Ph solution (G3.25-60) and 3 wt% gelatin/0.5 wt% Alginate-Ph solution after 60 min of exposure to the air containing H_2_O_2_ (G3.25-60 and G3.25/A0.5-60, respectively) were comparable to that of G3/HA0.5-60 (Figure 2b). G3.25-60 and G3.25/A0.5-60 hydrogels were prepared for evaluating the effect of the hydrogel stiffness and composition on HUVECs behavior shown in Section 3.3.

**Figure 5:**
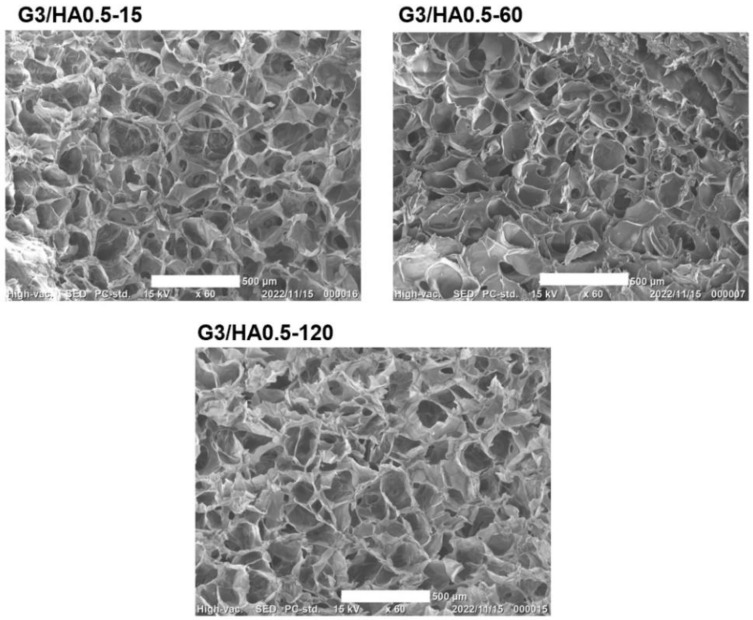
Scanning electron microscope observation of the cross-section of freeze-dried specimens of Gelatin-Ph/HA-Ph hydrogel obtained through 15–120 min H_2_O_2_ exposure time. Scale bar: 500 μm.

Figure 5 shows the cross-section images of freeze-dried G3/HA0.5 hydrogels observed using SEM. No remarkable difference in the pore size was found for the specimens obtained through different H_2_O_2_ exposure time. This result indicates that the difference in the microstructure in the hydrogels that caused the differences in mechanical property (Figure 2) and enzymatic degradation (Figure 3) resulting from the differences in H_2_O_2_-mediated cross-linking and degradation was not large enough to induce the difference in the ice nucleation and ice crystal growth during freezing the hydrogels. It has been reported that the ice nucleation and ice crystal growth during freezing of hydrogels govern the porous structure of the resultant dried specimens [34,35,36].

### 3.2. HUVECs Behavior on Hydrogels

We then evaluated the influence of the physicochemical property changes on the behavior of the HUVECs. Initially, we investigated the viability of the HUVECs on G3 and G3/HA0.5 hydrogels prepared by exposing air containing H_2_O_2_ for 15–120 min. Then, the hydrogels were seeded with cells after soaking overnight in a medium containing catalase to remove the remaining H_2_O_2_. HUVECs showed >90% viability on all the hydrogels independent of the composition and the exposure time. This result indicates that the hydrogels prepared through exposure to the air containing H_2_O_2_ have no cytotoxicity on HUVECs. 

**Figure 6:**
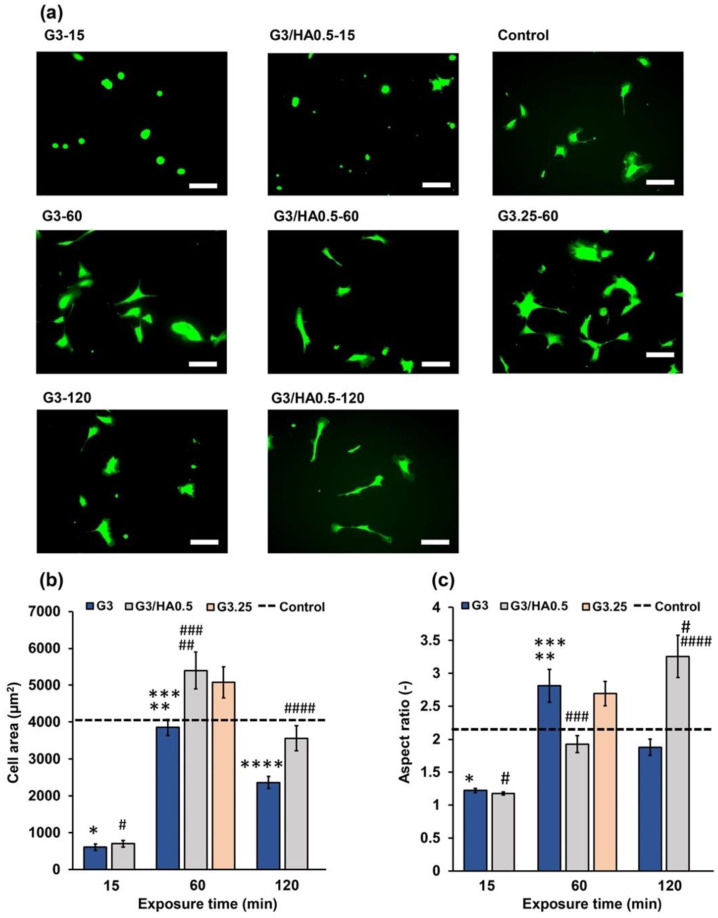
(**a**) Fluorescence micrographs of HUVECs stained with calcein-AM after 2 days of post culture on the G3, G3/HA0.5, and G3.25 hydrogels. HUVECs cultured on the polystyrene cell culture plate are considered as the control, and the dashed line indicates the corresponding values for the control. Effect of the exposure time to the air containing 16 ppm H_2_O_2_ on the (**b**) cell area and (**c**) aspect ratio of HUVECs on G3, G3/HA0.5, and G3.25 hydrogels. Scale bar:100 μm. Bars: S.E. (*n* ≥ 35 cells). * *p* < 0.01, ^#^ *p* < 0.01, 15 min exposure time compared to control (cell area); * *p* < 0.01, ^#^ *p* < 0.01, 15 min and 120 min exposure time compared to control (aspect ratio); ** *p* < 0.01, ^##^ *p* < 0.01, 15 min exposure time compared to 60 min (cell area); ** *p* < 0.01, 15 min exposure time compared to 60 min (aspect ratio); *** *p* < 0.01, ^###^ *p* < 0.01, 60 min compared to 120 min; **** *p* < 0.05, ^####^ *p* < 0.01, 15 min compared to 120 min (cell area); ^####^ *p* < 0.01, 15 min compared to 120 min (aspect ratio). Tukey HSD.

Cell area analysis showed that HUVECs cultured on G3 and G3/HA0.5 hydrogels had a stiffness-dependent alteration of the cell area. The cells on the G3-60 and G3/HA0.5-60 had the largest area (Figure 6a,b). Interestingly, cell elongation analysis based on the aspect ratio of the cells showed a different elongation trend of HUVECs cells on G3 and G3/HA0.5 hydrogels. While cells on G3 showed a stiffness-dependent elongation, in which cells cultured on G3-120 had a lower aspect ratio than those on G3-60 hydrogel, those on G3/HA0.5-120 hydrogel showed a higher aspect ratio on 120 min hydrogels (Figure 6c). This result is possibly mediated by the HA-Ph degradation in the 120 min exposed hydrogels. In fact, the cells on G3.25-60 hydrogel with a comparable stiffness with G3/HA0.5-60 (Figure 2b) showed a similar aspect ratio with those on G3-60 (*p* = 0.906, Tukey HSD) but a different aspect ratio with those on G3/HA0.5-60 (*p* = 0.020, Tukey HSD).

Previous studies have reported that the low-molecular-weight HA (LMW-HA) could induce cell elongation [37] and epithelial-to-mesenchymal transition (EMT) [38]. The EMT process involves a physiological transition marked by morphological changes to elongated spindle-like morphology. As shown in Figure 4 and Figure S2b, prolonged exposure to air containing H_2_O_2_ could degrade the HA-Ph. The degraded fragments of HA-Ph might interact with cell receptors that induce cell elongation [37]. In our previous study, similar result was obtained with mouse mammary cells cultured on the composite hydrogel (Gelatin-Ph/HA-Ph) obtained through 120 min of exposure to the air containing 16 ppm H_2_O_2_ [26]. In general, this result shows that HUVECs adhesion depends on the composition and the mechanical property of the hydrogel.

### 3.3. HUVECs Network Formation

**Figure 7:**
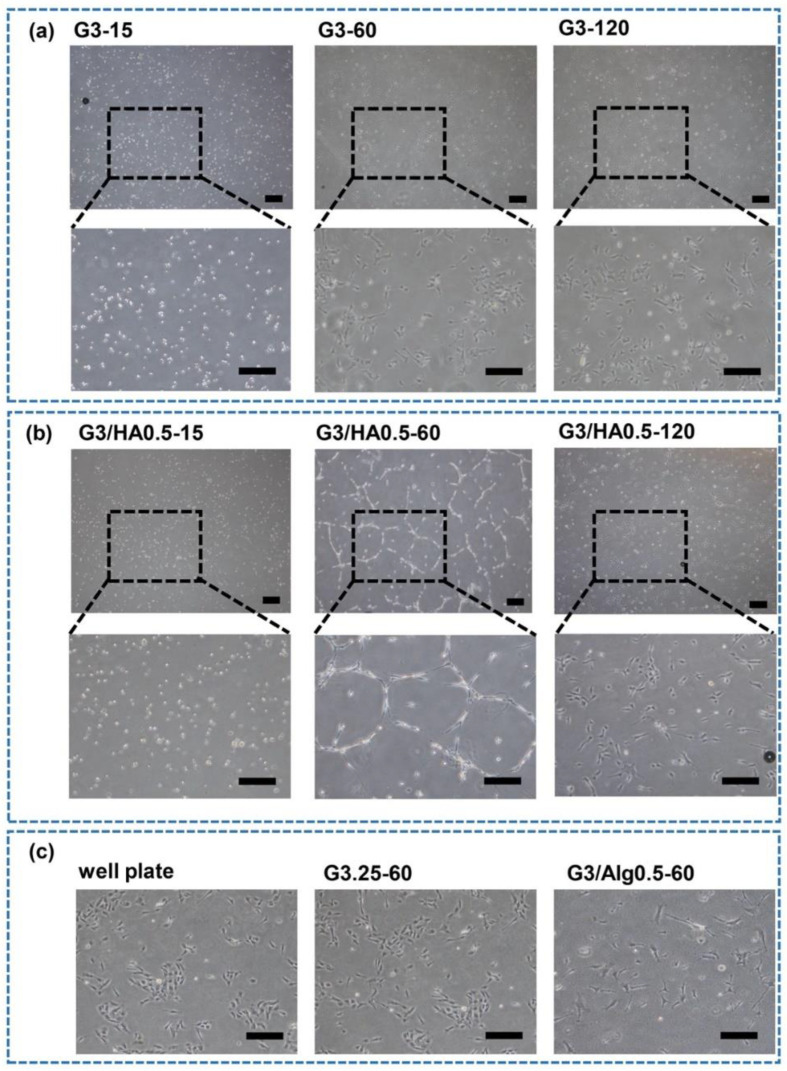
The effect of hydrogel composition and air containing H_2_O_2_ exposure time on HUVECs capillary-like network formation. HUVECs were seeded at 4.0 × 10^4^ cells/cm^2^ on (**a**) G3-15, G3-60, G3-120, (**b**) G3/HA0.5-15, G3/HA0.5-60, G3/HA0.5-120, and (**c**) G3.25-60, G3/Alg0.5-60, and culture well plate (controls). Scale bar: 100 μm.

Finally, we investigated the HUVECs network formation on each hydrogel by seeding HUVECs at 4.0 × 10^4^ cells/cm^2^. This seeding density was selected considering the successful network formation in previous studies [33]. As shown in Figure 7, HUVECs formed a visible network only on G3/HA0.5-60 hydrogel. The different behavior of the cells on G3/HA0.5-15, -60, and -120 hydrogels suggests the possible effect of hydrogel stiffness on the network formation. The different behavior of the cells on G3-60 and G3/HA0.5-60 also suggests the possible effect of the stiffness. G3/HA0.5-60 had the highest stiffness in G3 and G3/HA0.5 hydrogels (Figure 2a). However, the sole effect of the hydrogel stiffness on the network formation of HUVECs was denied by the non-network formations of the cells on G3.25-60 and G3/A0.5-60 hydrogels having similar stiffnesses with G3/HA0.5-60 (Figure 2b).

These results demonstrate that the combined effects of hydrogel stiffness and the low-molecular-weight HA-Ph generated through the degradation of HA-Ph by H_2_O_2_ caused the network formation of HUVECs on G3/HA0.5-60 hydrogel. The detailed mechanism of how the HA molecular weight and stiffness of the hydrogel affect the HUVECs behavior is unknown and will be a subject of our future study. However, there are several possible mechanisms. The presence of small fragments of HA, such as those as a product of HA-Ph degradation by air containing H_2_O_2_ exposure (Figure 4 and Figure S2), was reported to induce HUVECs capillary-like network formation by interacting with CD44 and RHAMM receptors [39,40]. In addition, HA-CD44 interaction could activate γ-adducin, which plays a role in the HUVECs tube formation [41]. Meanwhile, the interaction between HA and RHAMM induces AP-1 binding to the RHAMM promoter, promoting the capillary-like network formation of HUVECs [41,42].

In addition to the chemical signaling of ECM components, ECM stiffness plays an essential role in the capillary-like network formation of HUVECs through mechanical signaling. Joseph et al. reported the effect of substrate mechanics and matrix chemistry on endothelial cell network assembly using polyacrylamide substrates derivatized with type I collagen. They investigated that stiff hydrogel (2.5–10 kPa) with low cell-substrate adhesiveness could form a capillary-like network, while soft substrate (0.2–1 kPa) retained a round morphology without network formation [43]. Therefore, the combination of both substrate stiffness and ECM components is vital for the capillary-like network formation of HUVECs. Taken together, our studies demonstrate that the effect of H_2_O_2_ on inducing polymer crosslinking and degradation could modulate the behavior of HUVECs. From the translational perspective, these investigations are useful in various tissue engineering applications. Several studies have reported that the alteration in HA molecular weight and amount are characteristic of pathological conditions. The accumulation of HA in cancer stroma is a marker of malignancy for different kinds of tumors [44]. In addition, angiogenesis is essential for invasive tumor growth and constitutes a crucial point in controlling tumor progression [45]. In cancer therapy, inhibition of angiogenesis may be a valuable new approach. Therefore, our findings could be used as a tool for the in vitro study of cancer therapy through factors affecting inhibition and stimulation of angiogenesis.

## 4. Conclusions

In this study, we investigated the influence of the composition and mechanical properties of the hydrogels on the behavior of HUVECs. We tuned the degree of polymer degradation and hydrogel stiffness by simply controlling the exposure time to air containing H_2_O_2_ during the hydrogel preparation. HUVECs showed different responses depending on the degree of polymer degradation and the stiffness of the hydrogel. HUVECs cultured on Gelatin-Ph/HA-Ph hydrogel showed higher elongation on prolonged exposure to air containing H_2_O_2_, a phenomenon that was not observed in the absence of HA-Ph. More importantly, HUVECs network formation was observed only on stiff Gelatin-Ph/HA-Ph hydrogels, while cells on hydrogels composed of Gelatin-Ph alone or mixed with Alginate-Ph instead of HA-Ph showed no network formation regardless of the stiffness. Taken together, these results showed that HA-Ph addition to the Gelatin-Ph and the mechanical properties played an important role in governing the HUVECs behavior. Therefore, we believe that our findings could be useful for the fabrication of the hydrogels for the studies using HUVECs, including the biofabrication of artificial tissues and for in vitro studies to understand the factors that promote or inhibit angiogenesis.

## Figures and Tables

**Table 1:** The abbreviation of hydrogels and the H_2_O_2_ exposure time applied to fabricate the hydrogels from the solutions containing 1 U/mL HRP.

Abbreviation	Hydrogel Composition	H_2_O_2_ Exposure Time (min)
G3-15	3 wt% Gelatin-Ph	15
G3-60	3 wt% Gelatin-Ph	60
G3-120	3 wt% Gelatin-Ph	120
G3/HA0.5-15	3 wt% Gelatin-Ph/0.5 wt% HA-Ph	15
G3/HA0.5-60	3 wt% Gelatin-Ph/0.5 wt% HA-Ph	60
G3/HA0.5-120	3 wt% Gelatin-Ph/0.5 wt% HA-Ph	120
G3.25-60	3.25 wt% Gelatin-Ph	60
G3/Alg0.5-60	3 wt% Gelatin-Ph/0.5 wt% Alginate-Ph	60

## Data Availability

All data generated or analyzed during this study are included in this published article and its supplementary information files.

## Supplementary Materials

The following supporting information can be downloaded at: https://www.mdpi.com/article/10.3390/polym14225034/s1, Figure S1: UV–vis and ^1^H NMR spectroscopy of Gelatin-Ph; Figure S2: Intensity–time curve of (a) Gelatin-Ph and (b) HA-Ph after exposure to air containing 16 ppm H_2_O_2_ for 0–120 min.
