# Supplementary material for: Human Umbilical Vein Endothelial Cells Form a Network on a Hyaluronic Acid/Gelatin Composite Hydrogel Moderately Crosslinked and Degraded by Hydrogen Peroxide
Source: Polymers (Basel). 2022 Nov 20;14(22):5034. doi: 10.3390/polym14225034 (PMC9696239; doi:10.3390/polym14225034)
Supplement: Supplementary file 1 [file polymers-14-05034-s001.zip › polymers-2029027-supplementary.pdf]

polymers

Supplementary Information

Human Umbilical Vein Endothelial Cells Form a Network on a Hyaluronic Acid/Gelatin Composite Hydrogel Moderately Crosslinked and Degraded by Hydrogen Peroxide

Kelum Chamara Manoj Lakmal Elvitigala, Wildan Mubarok, and Shinji Sakai*

Department of Materials Engineering Science, Graduate School of Engineering Science, Osaka University, Toyonaka 560-8531, Japan
* Correspondence: sakai@cheng.es.osaka-u.ac.jp

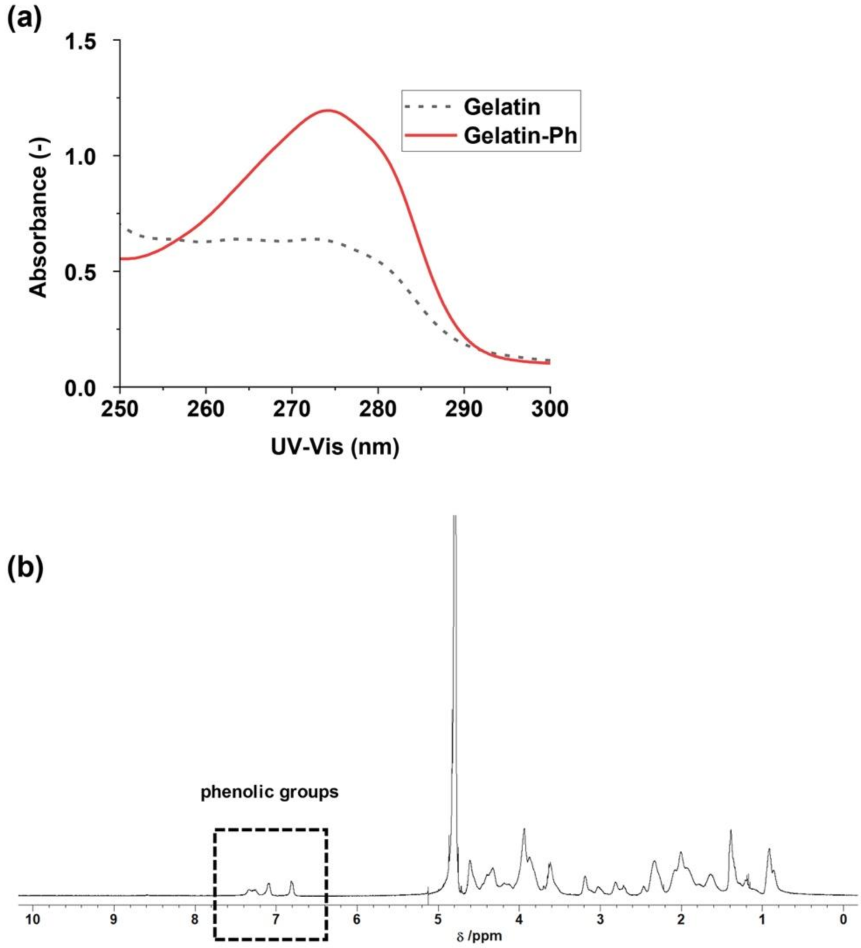

Figure S1. UV-Vis and ^{1}H NMR spectroscopy of Gelatin-Ph. (**a**) UV-Vis absorbance of unmodified gelatin and Gelatin-Ph. The absorbance peak at 275 nm indicated the presence of phenol groups in the Gelatin-Ph. **(b**) ^{1}H NMR spectroscopy of Gelatin-Ph (400 MHz, 25 °C, 64 scans) showing the presence of phenolic groups at 6.5 to 7.5 ppm.

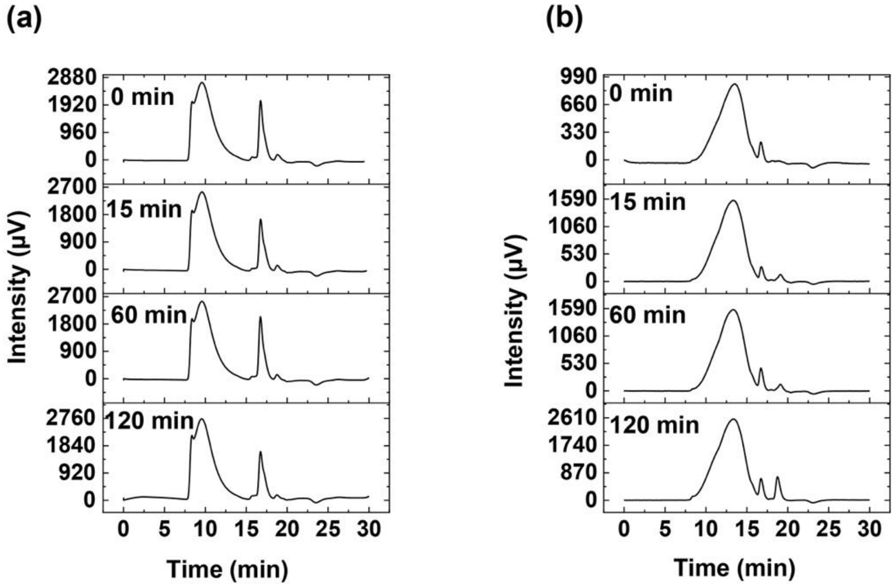

Figure S2. Intensity-time curve of (**a**) Gelatin-Ph and (**b**) HA-Ph after exposure to air containing 16 ppm H_2O_2 for 0 – 120 min. Peak top molecular weight relative to the polyethylene glycol (standard). LC conditions: separation was conducted on a SHIMADZU HPLC system equipped with Shodex OHpak SB804 HQ, 8 mmID x 300 mmL column; mobile phase PBS at pH 7.4; flowrate, 0.7 mL/min; temperature, 25 °C.
